# Cholera and Shigellosis: Different Epidemiology but Similar Responses to Climate Variability

**DOI:** 10.1371/journal.pone.0107223

**Published:** 2014-09-17

**Authors:** Benjamin A. Cash, Xavier Rodó, Michael Emch, Md. Yunus, Abu S. G. Faruque, Mercedes Pascual

**Affiliations:** 1 Center for Ocean-Land-Atmosphere Studies, Fairfax, Virginia, United States of America; 2 Institut Català de Ciències del Clima (IC3), Barcelona, Catalunya, Spain; 3 Institució Catalana de Recerca i Estudis Avançats, Barcelona, Catalunya, Spain; 4 University of North Carolina Chapel Hill, Chapel Hill, North Carolina, United States of America; 5 International Centre for Diarrheal Disease Research, Dhaka, Bangladesh; 6 Department of Ecology and Evolutionary Biology University of Michigan, Ann Arbor, Michigan, United States of America; 7 Howard Hughes Medical Institute, Chevy Chase, Maryland, United States of America; University of California, Merced, United States of America

## Abstract

**Background:**

Comparative studies of the associations between different infectious diseases and climate variability, such as the El Niño-Southern Oscillation, are lacking. Diarrheal illnesses, particularly cholera and shigellosis, provide an important opportunity to apply a comparative approach. Cholera and shigellosis have significant global mortality and morbidity burden, pronounced differences in transmission pathways and pathogen ecologies, and there is an established climate link with cholera. In particular, the specific ecology of *Vibrio cholerae* is often invoked to explain the sensitivity of that disease to climate.

**Methods and Findings:**

The extensive surveillance data of the International Center for Diarrheal Disease Research, Bangladesh are used here to revisit the known associations between cholera and climate, and to address their similarity to previously unexplored patterns for shigellosis. Monthly case data for both the city of Dhaka and a rural area known as Matlab are analyzed with respect to their association with El Niño and flooding. Linear correlations are examined between flooding and cumulative cases, as well as for flooding and El Niño. Rank-correlation maps are also computed between disease cases in the post-monsoon epidemic season and sea surface temperatures in the Pacific. Similar climate associations are found for both diseases and both locations. Increased cases follow increased monsoon flooding and increased sea surface temperatures in the preceding winter corresponding to an El Niño event.

**Conclusions:**

The similarity in association patterns suggests a systemic breakdown in population health with changing environmental conditions, in which climate variability acts primarily through increasing the exposure risk of the human population. We discuss these results in the context of the on-going debate on the relative importance of the environmental reservoir vs. secondary transmission, as well as the implications for the use of El Niño as an early indicator of flooding and enteric disease risk.

## Introduction

Comparative studies of associations between climate variability and human health across different infectious diseases are largely lacking. Potential climate influences are typically addressed only for specific infections and locations for which sufficiently long retrospective records exist. Such comparison studies, however, are instrumental in identifying systemic breakdowns in human health at the population level that do not depend on specific pathogen ecologies or epidemiology. Similarities in responses to climate forcing instead signal common mechanisms and, in so doing, identify key pathways of action for intervention. In addition, associations between disease risk and slowly varying features of the climate system allow for the possibility of developing early warning systems for disease risk in the affected areas.

Diarrheal illnesses provide one important disease class for the application of a comparative approach. Cholera, arguably the paradigmatic water-borne diarrheal disease, is caused by the bacteria *Vibrio cholerae* and has been responsible for numerous pandemics throughout history, including the current era [1]. Infection generally results from ingesting water contaminated with the bacteria, and left untreated leads to mortality rates as high as 50% [2]. Global mortality rates for cholera are estimated at ∼3% [3]. Mortality for shigellosis, another widely spread diarrheal illness that is caused by bacteria of the *Shigella* genus can reach 5–15% for some strains in areas with poor medical care [4]–[5]. The infectious dose for shigellosis is also substantially lower than for cholera and the disease is responsible for significant mortality and morbidity in the developing world [6]. Given their global mortality and morbidity burden, the potentially pronounced differences in transmission pathways and pathogen ecologies, and the existing evidence for a role of climate forcing on cholera, cholera and shigellosis represent excellent candidates for a comparative study on their associations with climate.

Endemic cholera has been prominent in studies on the influence of climate variability on diarrheal diseases. In particular, the El Niño-Southern Oscillation (ENSO), widely recognized as one of the most significant modes of interannual climate variability, has been shown to affect the severity of seasonal outbreaks in Bangladesh [7]–[12]. Favorable conditions for increased cholera risk in the fall have been shown to be associated with positive ENSO (El Niño) events in the preceding winter, thus establishing ENSO as a potential early-warning indicator for Bangladesh cholera outbreaks. At the local level increased rainfall and associated flooding have also been shown to increase fall cholera cases, and represent the means by which the remote influence of ENSO in transmitted to Bangladesh [11], [13].

The particular attention paid to cholera is due in no small part to the extended record of cases available for Bangladesh, advances on the microbial ecology of *Vibrio cholerae*, and the recognition that the pathogen survives outside the human host in aquatic environments such as estuaries and brackish water [14]–[16]. Climate conditions favoring the population growth or survival of the pathogen would exacerbate transmission through environmental aquatic reservoirs. This paradigm has led to an emphasis on mechanisms that are pathogen specific in mediating the effect of climate variability. An alternative, although not necessarily exclusive view, is one in which the vulnerability of the human host plays a key role and at seasonal and interannual time scales is modified by anomalous climate conditions such as extreme floods [17]–[19].

The relationship between water-borne bacterial diseases in Bangladesh and the monsoon rains is of particular interest, given that Bangladesh lies at the confluence of three major rivers (Ganges/Padma, Brahmaputra, Megna) in an extensive estuarine region that is considered the hearth of cholera. Bangladesh is also a low-lying country that experiences some of the highest rainfall totals in the Indian monsoon region and is inundated to a greater or lesser degree on an annual basis. While a certain degree of flooding is necessary to maintain the fertility of the delta system and associated agriculture, major flooding events have resulted in serious loss of life and destruction of infrastructure throughout Bangladesh's history [20]. Given the evidence linking flooding and post-monsoon cholera outbreaks [17]–[18], [22]–[24], it is of particular interest to consider what role the severity of annual flooding might play for shigellosis in that same region and season, particularly in the context of potential remote forcing by ENSO.

The extensive surveillance program of the International Center for Diarrheal Diseases Research, Bangladesh (icddr,b) makes it possible for us to revisit here the known associations between cholera and climate variability, and to specifically inquire about the possibility of similar but unknown patterns for shigellosis. In the results presented below, we show that there are indeed similar associations between cholera and shigellosis cases in Bangladesh and flooding, as well as for cholera and shigellosis cases and sea surface temperatures in the tropical Pacific. These similar associations have significant implications for the mechanisms behind the influence of climate on enteric disease outbreaks in Bangladesh, as well as for the use of ENSO as an early warning indicator of outbreak risk.

We indeed find a strong similarity of association patterns for post-monsoon outbreaks, whose implications for the mechanisms behind the influence of climate forcing we discuss.

## Data and Methodology

### Ethics Statement

The Diarrheal Disease Surveillance System (DDSS) of icddr,b is a routine ongoing activity of the Dhaka and Matlab Hospital, which has been approved by the Research Review Committee (RRC) and Ethical Review Committee (ERC) of icddr,b. At the time of enrollment, verbal consent was taken from the adult patients and caregivers or guardians in case of the children patients, with the information to be stored in the hospital database and used for conducting research. This verbal consent was documented by keeping a check mark in the questionnaire, which was again shown to the adult patients or the parents/guardians of children patients. Patients or parents/guardians were assured about the non-disclosure of information collected from them, and were also informed about the use of data for improving patient care activities as well as scientific research and publication without disclosing the name or identity of the patients. ERC was satisfied with the voluntary participation, maintenance of the rights of the participants and confidential handling of personal information by the hospital physicians and has approved this consent procedure.

### Data

Flood Affected Area (FAA), expressed as the percentage of the total area of Bangladesh inundated, was obtained from the annual reports of the Bangladesh Flood Forecasting and Warning Center (FFWC; http://www.ffwc.gov.bd). Values range from 0.2% in 1994 to a remarkable 68% during the severe flooding of 1998. Although FAA is only available as annual values, flooding in Bangladesh is dominated by the rainy season (June-September; JJAS). The reported FAA for each year was taken to be representative of this period.

The NINO34 index is a standard and widely used measure of the strength and state of ENSO, the dominant mode of climate variability on interannual timescales. Values were provided by the National Oceanic and Atmospheric Administration (NOAA) Climate Prediction Center. NINO34 is defined as the area-averaged sea surface temperature (SST) over the region (5°S-5°N, 120°W-170°W) and is presented here as 3-month running mean anomalies calculated relative to the 1971-2000 base period. Gridded SST values used to calculate the rank correlation maps (see below) were taken from the Hadley Centre Sea Ice and Sea Surface Temperature (HadISST) v1.1 data set [25].

The disease data analyzed in this work was taken from two surveillance programs overseen by the icddr,b. In one program, based in icddr,b′s Dhaka Hospital, stool specimens from every 25^th^ patient were tested for cholera and shigellosis from 1979–1995, and from every 50^th^ patient from 1996 onwards. The second surveillance program is located in Matlab, Bangladesh, in a rural area approximately 57 km southeast of the capital of Dhaka. In contrast to the Dhaka hospital, patients attended to at the Matlab hospital are mostly those presenting symptoms of severe diarrheal illness. Stool specimens of all diarrhea patients from the Health and Demographic Surveillance System (HDSS) area were tested for shigellosis and cholera. In both locations cases were separated by causative species and strain, and in the analysis presented here we focus exclusively on cases due to the dominant form of the each disease for our period of study, which is 1983–2010. For cholera this was this El Tor biotype, which replaced the former Classical biotype in the past decades and is now the dominant strain both in Bangladesh and around the world. For shigellosis *S. flexneri* represent the dominant form of the disease in Bangladesh.

**Figure 1:**
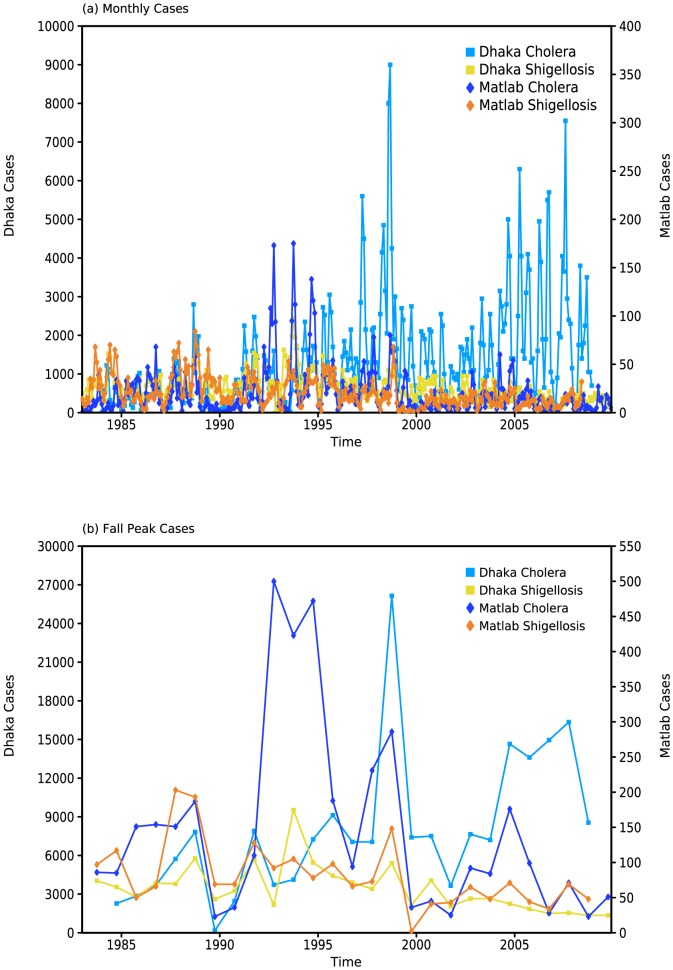
Time series of (a) monthly *S. flexneri* shigellosis and *V. cholerae* El Tor cholera cases and (b) cases during the fall peak (August-December; ASOND) from 1983–2009 for Dhaka and Matlab.

Time series of the monthly cases (Fig. 1a) and the magnitude of the fall peak (Fig. 1b) for the two diseases in both Matlab and Dhaka show that, in addition to significant interannual variability for both diseases and locations, cholera cases exceed those of shigellosis and that there is a clear downward trend in Dhaka shigellosis cases for this period.

### Methodology

Our focus in this work is on interannual variations in disease outbreaks, as opposed to longer-term trends, and as such all data was detrended prior to analysis. As a check on the sensitivity of our results to the removal of the long-term trends we first analyzed data detrended using a simple linear regression. We then repeated the analysis using data detrended via Singular Spectrum Analaysis (SSA), which allows for a more flexible and nonlinear definition of the trend [26]. Our results were not sensitive to the choice of detrending technique (See File S1) and as such results reported here are based on the simpler linear method.

To quantify the magnitude and geographical extent of the link between the occurrence of water borne disease cases and potential environmental drivers, such as SST, we calculate Spearman rank-correlations between reported cases, flooding, and SST (see also [11], [27]). We employed rank-correlation, rather than the more common Pearson's correlation, because it is a non-parametric method more suitable for use with the non-normally distributed flooding and disease data. The rank-correlation, ρ, is calculated as
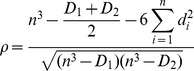
(1)in the case where the data includes ties [28], where *n* is the number of months, *d_i_* is the difference in rank for each month, and *D_1_* and *D_2_* are the sum of the cubes of the sizes of the ties in the first and second samples, respectively, where the size of a tie is defined as the number of months with the same value. For *n*>20 we can test for the significance of the above quantity using the transformed, normally distributed variable



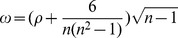
(2)


## Results and Discussion

Time series of monthly cases (Fig. 1a) and the magnitude of the fall peak (Fig. 1b) of cholera and shigellosis in Matlab and Dhaka show that, in addition to the significant interannual variability for both diseases and locations, there is a downward trend in Dhaka shigellosis cases for this period that is significant at the 95% level (as determined by SSA; see File S1 for details). The significance of the trend is further emphasized by the fact that only for Dhaka shigellosis do results made using the detrended and raw case data differ.

As noted in the Introduction, previous studies have identified a clear statistical association, as well as a physical link, between post-monsoon cholera cases in Matlab and ENSO events in the preceding winter. Building on this previous work (e.g., [11]–[12]), we calculate the rank correlation between shigellosis cases during the ‘fall peak’ (August-December; ASOND) and SST at every grid point for the preceding boreal winter (December-February; DJF). Calculating the correlation for each grid point, rather than for a single index defined for a limited and predetermined region, allows for a fuller description of the association between disease outbreaks and global SST. Calculating correlation maps for multiple diseases and locations allows for a richer comparison of their associations with SST as well.

**Figure 2:**
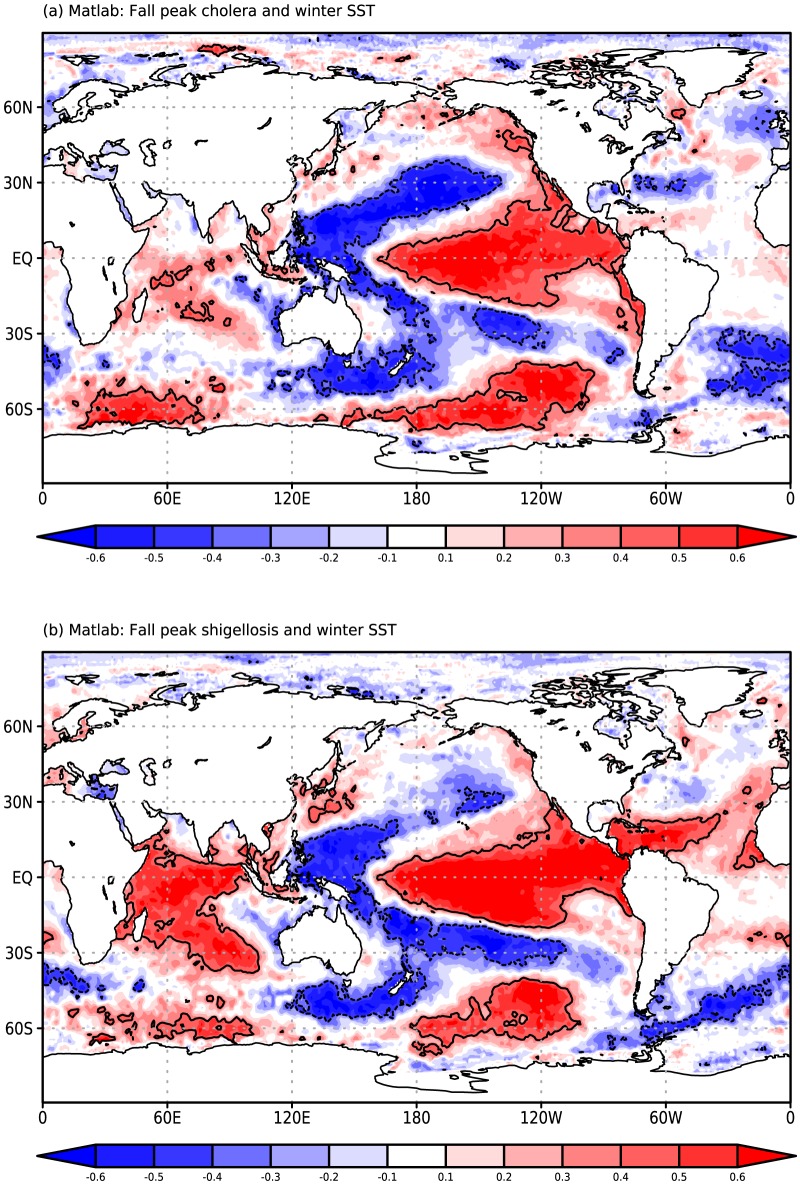
Rank-correlation maps for Matlab (a) fall peak (August-December; ASOND) El Tor cholera and (b) ASOND *S. flexneri* shigellosis cases and preceding December-February sea surface temperature (SST). Maps are correlated at a value of 0.88 for the subregion (35S, 35N).

The correlation map for ASOND cholera in Matlab and DJF SST (Fig. 2a) produces a pattern that closely resembles the one for Matlab shigellosis (Fig. 2b), particularly through the tropical regions. This visual similarity is confirmed by the very high pattern correlation of 0.88 between the two maps. While there are minor differences between the patterns for cholera and shigellosis in the Indian Ocean, the diagnosed relationship with the tropical Pacific is nearly identical. Moreover, the maps also closely resemble the pattern of SST anomalies that mark the mature phase of a warm ENSO event, and both series are significantly correlated with the NINO34 index (0.42 for cholera, 0.55 for shigellosis; both p<0.05). This apparent association with El Niño was explored previously for Matlab cholera [11], in which experiments with a general circulation model confirmed the physical significance of the correlation pattern and its correspondence to ENSO. This analysis was essential in establishing that the patterns identified by the rank-correlation analysis do indeed represent ENSO, and do not resemble the ENSO pattern merely by chance. The close resemblance between the correlation patterns for cholera and shigellosis strongly implies that shigellosis is responding in a similar manner to the remote forcing from ENSO.

**Figure 3:**
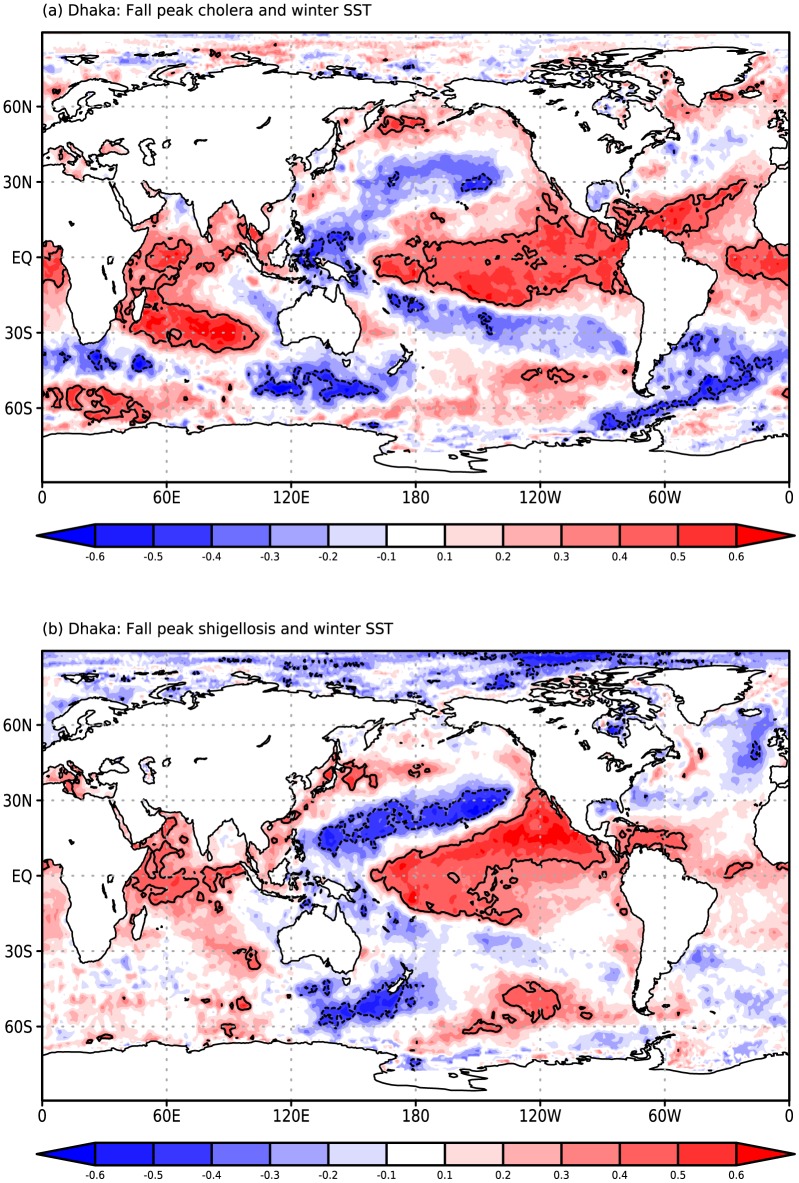
Rank-correlation maps for Dhaka (a) fall peak (August-December; ASOND) El Tor cholera and (b) ASOND *S. flexneri* shigellosis cases and preceding December-February sea surface temperature (SST). Maps are correlated at a value of 0.72 for the subregion (35S, 35N).

Applying this same correlation map analysis to Dhaka cholera (Fig. 3a) and shigellosis (Fig. 3b) cases, we again find strong similarities in the association patterns for the two diseases. While the Dhaka cholera map closely resembles the two Matlab maps, the highest correlations values are shifted towards the central Pacific for shigellosis. This shift results in a lower correlation between the two Dhaka patterns (0.72) than we find for Matlab, and a lower value of the correlation with the NINO34 index (0.54 for cholera, 0.21 for shigellosis; cholera correlation p<<0.01, shigellosis correlation not significant).

The fact that Dhaka shigellosis shows a statistically significant correlation with SST across a broad section of the tropical Pacific, while at the same time showing a relatively low correlation with the NINO34 index, highlights the advantages of analyzing correlation patterns rather than focusing on single indices. This shift in the region of significant correlations between Dhaka shigellosis and SST also suggests a closer association with the central, rather than eastern, Pacific component of ENSO. Thus, despite the dramatic differences in ecology between the two pathogens and the environments of the two study sites, we find a clear association between post-monsoon cases and ENSO in the preceding winter for both diseases and locations.

**Figure 4:**
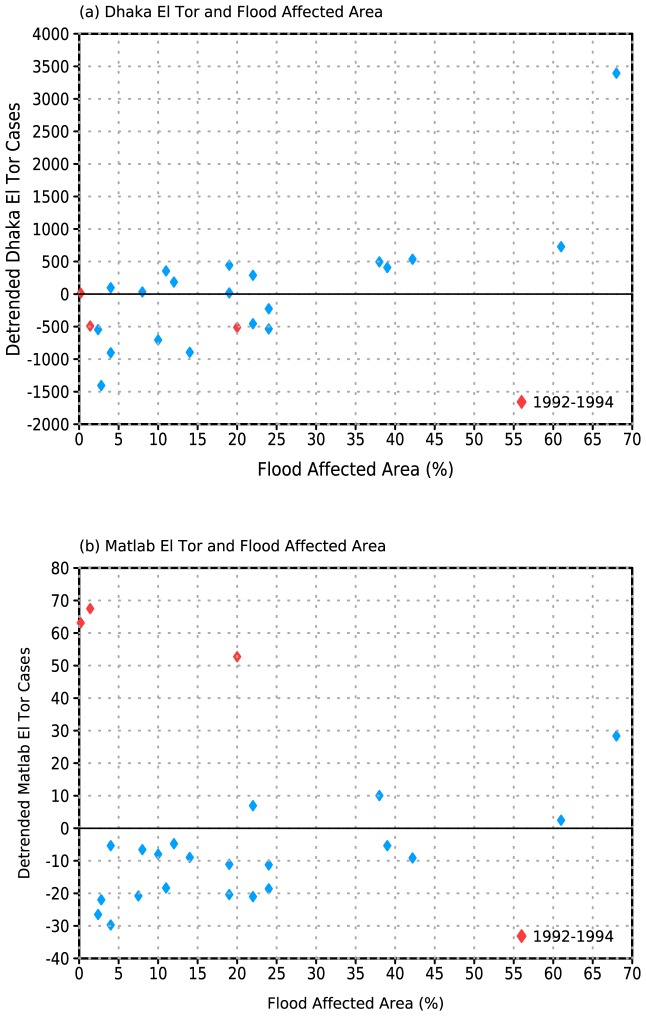
(a) Flood Affected Area (FAA) and linearly detrended fall peak (August-December; ASOND) Matlab El Tor cholera cases. Rank correlation of 0.56 when 1992–1994 removed. (b) FAA and linearly detrended ASOND Dhaka El Tor cholera incidence. Rank correlation of 0.74.

**Figure 5:**
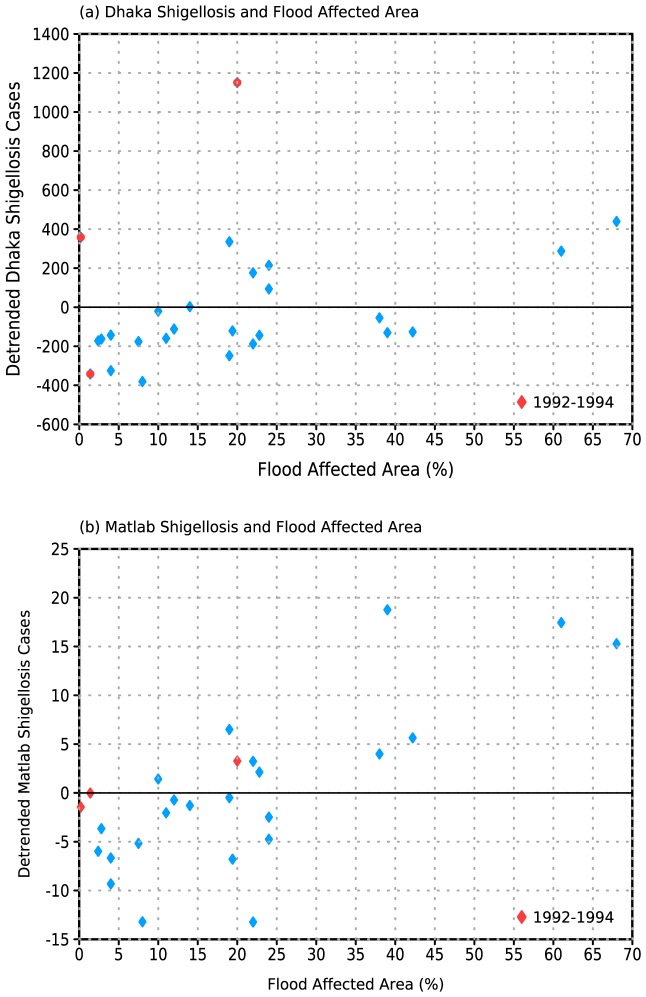
(a) Flood Affected Area (FAA) and linearly detrended fall peak (August-December; ASOND) Matlab *S. flexneri* shigellosis cases. Rank correlation of 0.74. (b) FAA and linearly detrended ASOND Dhaka *S. flexneri* shigellosis incidence. Rank correlation of 0.50.

Earlier work (e.g., [11]–[12]) advanced the theory that enhanced local rainfall and subsequent flooding is the mediating factor between tropical SST anomalies and cholera cases in Bangladesh. Enhanced local rainfall linked to remote SST anomalies has also been associated with increased malaria cases in northwest India [27]. Consistent with this mechanism, we find strong positive associations between FAA and both cholera (Fig. 4) and shigellosis (Fig. 5) cases in both locations. The relationship is particularly clear for the Dhaka fall peak in cholera cases (Fig. 4a), with no notable outliers and a rank correlation coefficient of 0.74 (p<<0.01). In contrast, for the Matlab data the period from 1992–1994 stands out sharply as an outlier, with 2–3 times the number of cases compared to other years with similar FAA. If this period is excluded the rank correlation coefficient with FAA is 0.54 (p<0.01; correlation is negligible if 1992–1994 are included). We do not find similar elevated values for this period in Dhaka. In addition, while 2007 was a year of heavy flooding and increased cholera prevalence in Dhaka, cases are relatively low in the Matlab record.

It is not known whether the inconsistencies in the cholera records for the two locations reflect sampling differences between the large, urban environment of Dhaka and the smaller, more rural environment of Matlab, or if they reflect a degree of localization in outbreaks. One difference in the sampling methodology is that in Matlab only patients who come from the Health and Demographic Surveillance System (HDSS) area are included in the study, while in Dhaka any patient who arrives at the hospital is included. It should also be noted that 1992–1994 is the period in which 0139 Bengal emerged in Bangladesh, and this may be related to the unusual values from Matlab.

For shigellosis there is somewhat greater scatter at lower levels of FAA (Fig. 5) than for cholera, perhaps reflecting the fact that shigellosis requires a much smaller infectious dose than cholera [6]. Overall the correlation with FAA is comparable to that of cholera (0.74 and 0.50 for Matlab and Dhaka, respectively, p<<0.01). Interestingly, the 1992–1994 period is unremarkable for shigellosis in Matlab but is a partial outlier for Dhaka (note that correlation between Dhaka shigellosis and NINO34 increases from 0.21 to 0.41 if these years are removed), further suggesting that enteric disease in Bangladesh during these three years merits additional investigation.

**Figure 6:**
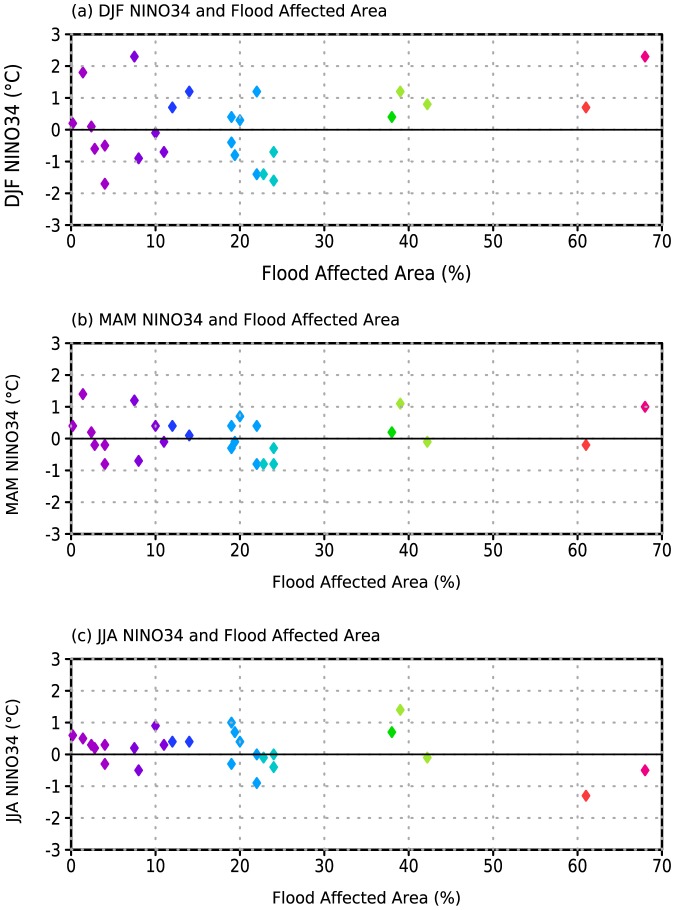
Flood Affected Area (FAA) versus (a) December-February (DJF) NINO34 index, (b) March-May (MAM) NINO34 index, and (c) June-August (JJA) NINO34 index.

The above analysis establishes that the annual flood affected area provides a clear predictor for the number of diarrheal disease cases in Bangladesh during the fall peak. However, it does so at very short leads. The fall peak (ASOND) overlaps with the end of the monsoon period (JJAS), providing only limited advanced warning for public health officials. In order to increase the lead-time of risk forecasts, we further explore the link between disease cases and ENSO (Figs. 2 and 3) in light of the association between disease cases and flooding (Figs. 4 and 5). Years with FAA below 30% are not strongly associated with the value of the NINO34 index (Fig. 6) for any of the three preceding seasons considered (December-January-February, DJF; March-April-May, MAM; June-July-August, JJA). FAA values above 30%, however, are not seen following negative DJF NINO34 values (Fig. 6a). For MAM (Fig. 6b) and JJA (Fig. 6c), these more severe floods can be associated with either positive or negative values of the NINO34 index. Hence, ENSO indices for the spring and summer seasons cannot be used to discriminate between strong and weak flooding years and thus high and low risk disease seasons.

## Summary and Conclusions

Cholera and shigellosis are two prominent diarrheal illnesses that together are responsible for significant mortality and morbidity throughout the developing world, including Bangladesh. The causative organisms, *V. cholerae* and *S. flexneri*, differ significantly in their ecology, transmission pathways, and infectious doses, among other features. Despite these differences, our analysis of case data taken from two separate surveillance sites in Bangladesh demonstrates that interannual variations in the severity of the fall outbreaks of both diseases are closely and similarly associated with the areal extent of the annual monsoon floods. Of potentially greater significance, particularly from the perspective of risk forecasting, we also find that both the monsoon floods and post-monsoon disease outbreaks are significantly correlated with ENSO activity in the preceding winter.

This association between winter ENSO and Bangladesh flooding is consistent with previous work [29], which identified a linear relationship between flooding and DJF NINO34 values. However, that study did not address the asymmetry between positive and negative ENSO events, or the variations in flooding between positive events. Recent work [30] suggests that the lag of two seasons between ENSO during DJF and the summer monsoon flooding is related to the excitation of a coupled air-sea mode of variability in the Indian Ocean region, which allows the impact of DJF ENSO anomalies to persist into summer. It is interesting to note that the magnitude of the flooding is not strongly correlated with the magnitude of the index, indicating that more than just ENSO is playing a role.

The striking similarity in the association between cholera and shigella and climate variability, despite the significant differences in the ecology of the two pathogens, strongly suggests that the two diseases share a similar transmission pathway in the post-monsoon period. Flooding inevitably increases the potential for exposure to contaminated water, an increase in risk likely to be exacerbated by the attendant overcrowding and breakdown in sanitary infrastructure. Flooding also, inevitably, subjects individuals to heightened stress, which is known to affect immune response and increases susceptibility to infectious disease [31]. While the relationship between flooding and El Tor cases in Bangladesh has been noted before (e.g., [13], [22]–[23], as has as an increase in shigellosis for the large flooding events or 1988, 1998 and 2004 [17], this general association between flooding, cholera, and shigellosis has not been explored previously.

The association between flooding and shigellosis, in conjunction with the association between flooding and cholera is of particular interest because it demonstrates that the association between post-monsoon cholera cases and flooding is not unique. Rather it suggests that it is the general increase in exposure risk of the human population that follows flooding, rather than the specific ecology of any one pathogen, which underlies the impact of climate variability on diarrheal disease in Bangladesh. In particular our analysis demonstrates that a long-lived environmental reservoir connected to primary transmission, which is known to exist for cholera but not for shigellosis, is not a necessary condition for flooding to drive post-monsoon disease cases in. It instead suggests that climate variability acts by modulating secondary transmission, where the latter can involve different pathways, through water or food or person to person contact, and is characterized by a dependency on previous levels of infection in the population [16].

Our findings have focused on the post-monsoon season in part because of the established role of ENSO during the preceding winter on cholera during this part of the year, through the pathway of increased precipitation and flooding. It is worth mentioning however that the disease exhibits an additional, pre-monsoon, peak and the overall seasonal pattern is known to vary regionally, in particular with respect to the relative importance of the two seasons [22], [24]. Comparisons with other diarrheal diseases at this regional level would be informative. Similarly, comparison of spatio-temporal patterns within a large urban environment such as Dhaka would further elucidate similarities and differences in epidemiology, especially given the reported heterogeneity within the city itself in the response to ENSO [18].

Linking cholera and shigellosis to flooding provides a physical mechanism for environmental influence on the interannual variability of the two diseases; linking flooding to ENSO provides a potential mechanism for forecasting risk. Our analysis shows that the most severe flooding events in Bangladesh follow warm winter conditions in the central and eastern tropical Pacific; none follow cold conditions. This relationship allows for an initial prediction of flooding and disease risk to be made as early as the end of the boreal winter; the risk of severe flooding and associated disease outbreaks should be substantially reduced when ENSO is in a negative state. Understanding why severe flooding follows warm winter Pacific SST in only a subset of years, as well as why the relationship is not linear with the magnitude of the SST anomalies is critical to improving the accuracy of disease risk forecasts, and is the subject of ongoing research. Likewise, expanding comparative analysis to include other water-borne and vector-borne infections should further refine our understanding of the fundamental processes underlying the connections between climate variability and disease outbreaks.

## Supporting Information

File S1 **Sensitivity to choice of detrending method.**
(DOCX)
