# Supplementary material for: Cholera and Shigellosis: Different Epidemiology but Similar Responses to Climate Variability
Source: PLoS One. 2014 Sep 17;9(9):e107223. doi: 10.1371/journal.pone.0107223 (PMC4168003; doi:10.1371/journal.pone.0107223)
Supplement: File S1 — Sensitivity to choice of detrending method. (DOCX) [file pone.0107223.s001.docx]

Supplemental Material

Cholera and shigellosis: different epidemiology but similar responses to climate variability

Benjamin A. Cash, Xavier Rodó, Michael Emch, Mohammed Yunus, Abu Sayed Golam Faruque, and Mercedes Pascual

Singular spectrum analysis (SSA) is a technique for decomposing a time series into its constituent components according to its structure in the frequency domain. Specifically, SSA partitions variability contributions into orthogonal components and thus captures independent portions of the variability that can then be used for reconstructing a signal [1-2]. Frequency components are only counted once for reconstructions even if they project over different timescales, which can be a problem for simple spectral methods that do not control for phase differences and harmonics.

SSA has been effectively applied previously for signal extraction in time series of cholera incidence, as well as other diseases [3-4]. In the case of the Shigellosis data series analyzed here, we employ different recurrent SSA decompositions to maximize trend extractions. The embedding dimension *M* varies from longer (100<*M*<80) to shorter (*M*=40) window lengths, allowing for an effective reconstruction of the trend or low-frequency component while enabling an accurate reconstruction of the sources of interannual (IA) variability

Comparing correlation maps computed with linearly detrended data and the reconstructed interannual residual for Matlab and Dhaka shigellosis (Fig. S1), we find that while magnitudes of the Dhaka correlations are somewhat less for the SSA detrending (compare to Fig. 3b), the association between shigellosis and DJF ENSO is apparent for both methods. The Dhaka shigellosis trend was determined to be significant at the 95% level against null hypotheses of both white and red noise.

**References**

1. Vautard, Singular spectrum analysis in nonlinear dynamics, with applications to paleoclimatic time series. [*Physica D: Nonlinear Phenomena*](http://www.sciencedirect.com/science/journal/01672789) [**35**](http://www.sciencedirect.com/science/journal/01672789/35/3):395–424

2. Golyandina N, Nekrutkin V. Zhigljavsky A. 2001. Analysis of Time Series Structure: SSA and Related Techniques. Chapman & Hall/crc, 2001.

3. Rodó X, Pascual M, Fuchs G, Faruque ASG. 2002. ENSO and cholera: a nonstationary link related to climate change? *PNAS* **99**:12901-12906.

4. Pascual M, Ahumada J, Chaves LF, Rodó X, Bouma M. 2006. Malaria resurgence in the East African highlands: Temperature trends revisited *PNAS* **103**:5829-5834; doi:10.1073/pnas.0508929103 [Online 29 March 2006]


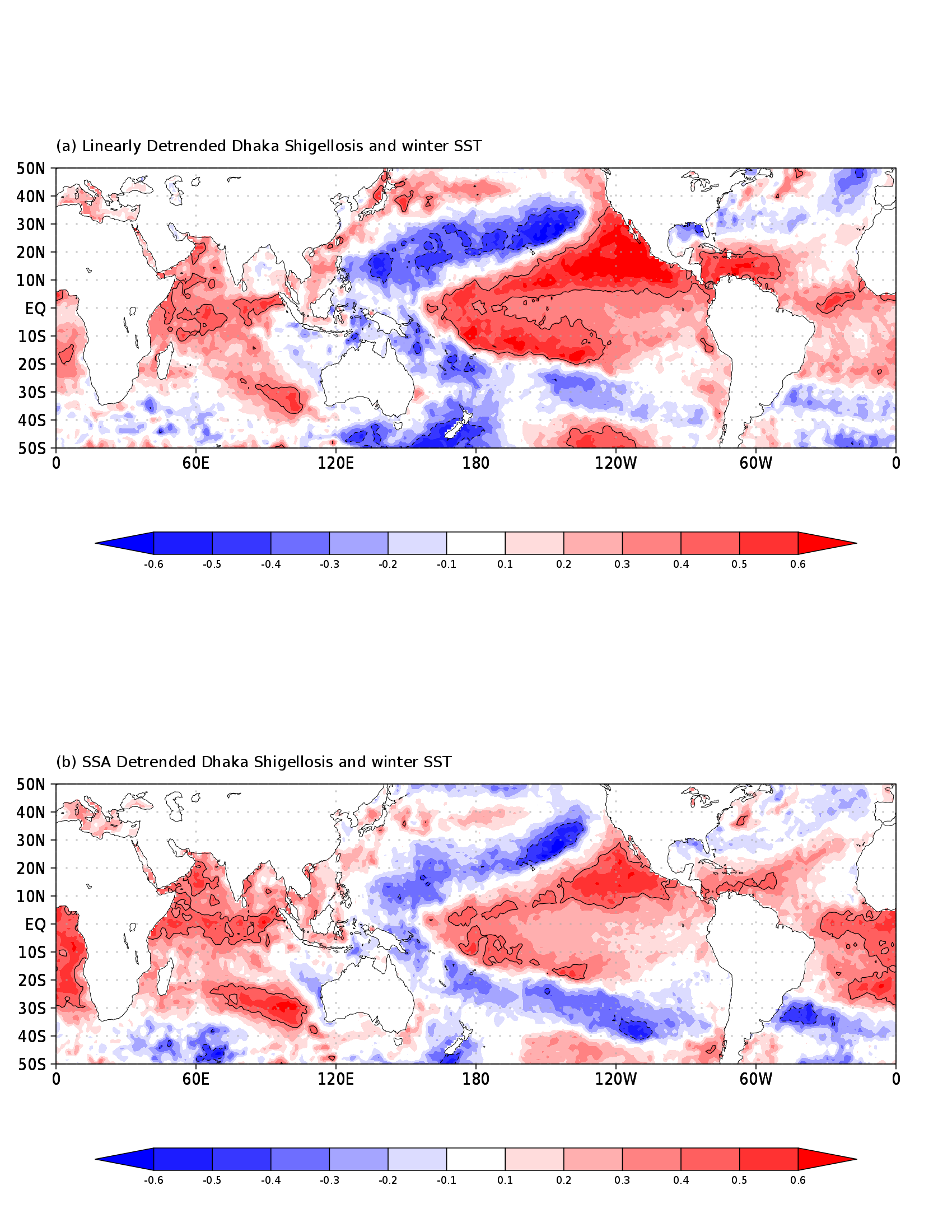
Figure S1: Rank correlation with DJF SST for (a) Linearly and (b) SSA detrended Dhaka shigellosis cases.
